# Editorial: VR-haptic technologies and their applications in dental education

**DOI:** 10.3389/froh.2026.1782946

**Published:** 2026-01-28

**Authors:** Sompop Bencharit, Barry Quinn, Reinhard Chun Wang Chau

**Affiliations:** 1 Haptics and Artificial Intelligence in Dental Education Network (HAIDEN); 2 Adams School of Dentistry, University of North Carolina at Chapel Hill, Chapel Hill, NC, United States; 3 School of Dentistry, University of Liverpool, Liverpool, United Kingdom; 4 Faculty of Dentistry, The University of Hong Kong, Hong Kong, Hong Kong SAR, China

**Keywords:** artificial intelligence (A.I.), dental education, education technology (EdTech), HAIDEN, haptic technology, international collaboration, simulation-based learning (SBL), virtual reality

## Introduction

The integration of virtual reality (VR) combined with haptic feedback into dental education represents one of the most promising developments in health professions training (1). These technologies offer repeatable, risk-free environments for deliberate practice, immediate objective feedback, and the simulation of tactile sensations essential to clinical procedures (2). As dental curricula worldwide face increasing demands for efficiency, standardization, and resource conservation, VR-haptic systems have emerged as powerful tools to bridge preclinical and clinical learning (3).

This Research Topic brings together eight studies that collectively provide robust empirical evidence on the efficacy, perceptions, and implementation of VR-haptic technologies across core areas of dental education. Many contributions stem from the collaborative efforts of international researchers within networks such as the Haptics and Artificial Intelligence in Dental Education Network (HAIDEN), reflecting a shared commitment to evidence-based innovation.

## Contributions to this research topic

The articles address three overlapping themes: skill acquisition in operative procedures, professional and faculty perceptions, and broader educational and cognitive impacts.

Several studies examine the direct impact of VR-haptic simulation on psychomotor skill development in operative dentistry. Investigations into cavity preparation in conservative dentistry and preclinical endodontic access cavities demonstrate that haptic-guided virtual training significantly improves precision, speed, and learner confidence compared with traditional methods Arroyo Bote et al. and Deeb et al. A randomized controlled trial exploring haptic-reinforced VR for pulpotomy training further reveals enhanced skill acquisition, more positive student perceptions, and measurable educational benefits, suggesting that prior simulation can meaningfully accelerate competence in pediatric procedures Florencia et al.

Applications extend beyond operative techniques. One contribution gathers insights from dental professionals on the use of VR-haptic simulators in periodontics, highlighting practical advantages for scaling, root planing, and instrumentation training Florencia and Flacco. Complementing these procedure-specific studies, a pilot investigation using functional neuroimaging (swLORETA qEEG) provides preliminary neurological evidence that 3D VR-haptic training positively influences cognitive functions associated with spatial awareness and procedural learning Manchorova et al.

A second major theme concerns perceptions, adoption patterns, and long-term evaluation. A decade-long comparative analysis of the widely used Simodont® Dental Trainer offers a valuable longitudinal perspective on system performance, user satisfaction, and evolving expectations Bakr et al. Faculty perspectives on integrating haptic simulation into curricula emphasize both opportunities and practical considerations, including curriculum design and instructor training Hashem et al. The collection concludes with results from a global education survey on VR-haptics adoption, revealing geographic variations, perceived barriers (e.g., cost, technical support), and facilitators of successful implementation Bencharit et al.

## Broader implications and challenges

Taken together, these eight articles strengthen the evidence base supporting VR-haptic technologies as effective adjuncts—or in some cases, potential replacements—for traditional preclinical training modalities (4). Typical findings include improved psychomotor outcomes, high learner and faculty acceptance, standardized assessment capabilities, and reduced consumption of physical resources such as extracted teeth and consumable materials. The inclusion of randomized trials, neuroimaging data, and large-scale survey results elevates the discourse beyond anecdotal enthusiasm toward rigorous validation.

These insights align with broader syntheses in the field, which highlight both the transformative benefits and implementation challenges of haptic-enhanced VR training, including faculty development needs and strategies to optimize preclinical assessment for handpiece-naïve students (1, 5).

Nevertheless, the studies also candidly address persistent challenges: high initial costs, the need for sustained technical infrastructure, faculty development requirements, and the need for further longitudinal research to confirm the transfer of skills to clinical performance. These balanced perspectives underscore that successful integration demands thoughtful curricular planning and institutional commitment.

## Future directions

The contributions in this Research Topic highlight several priority areas for continued investigation: optimization of haptic feedback fidelity, artificial intelligence(AI)-driven adaptive learning pathways, broader validation across dental specialties, and strategies to improve accessibility in resource-limited settings (6–9). Recent commentaries pose critical questions—such as whether digital gadgets can redefine the future of dental education—while emphasizing the role of international collaboration in shaping innovative, evidence-based approaches (10–12). Emerging work on AI applications, such as chatbot-based evaluation of knowledge in dentistry and mHealth tools for oral health monitoring, suggests potential synergies for AI-enhanced personalization and assessment within VR-haptic platforms (13–16).

Ongoing efforts, including the establishment of dedicated VR-haptic research groups and the publication of multinational papers on enhancing dental education through these technologies, will be essential to generate the multicentre, comparative data needed to inform policy and curriculum standards worldwide (12, 17).

## Conclusion

This collection marks an important milestone in documenting the maturation of VR-haptic technologies within dental education. By providing diverse methodological approaches—from controlled trials and neuroimaging to global surveys—these articles collectively affirm the transformative potential of haptic-enhanced simulation while maintaining critical scrutiny of its limitations. As dental educators embrace an increasingly digital future—“thinking big” through collaborative networks like HAIDEN—evidence such as that presented here will guide the thoughtful, equitable adoption of these technologies to produce more competent and confident practitioners ultimately.
